# Evaluation of blood type as a potential risk factor for early postpartum hemorrhage

**DOI:** 10.1371/journal.pone.0214840

**Published:** 2019-04-04

**Authors:** Mais Ali-Saleh, Ofer Lavie, Yoram Abramov

**Affiliations:** Department of Obstetrics and Gynecology, the Lady Davis Carmel Medical Center, Technion University, Rappaport Faculty of Medicine, Haifa, Israel; University of Washington, UNITED STATES

## Abstract

**Objective:**

Studies have demonstrated an association between ABO blood type and bleeding status. The aim of this analysis was to determine whether O blood type is associated with higher early postpartum hemorrhage (PPH) risk as compared to other blood types.

**Study design:**

In this retrospective case-control study, data was gathered form 4,516 deliveries occurring at our institution between 2014 and 2016. Cases were categorized into one of two groups according to women’s major blood type (O or non-O), and thereafter according to minor blood type (RH positive or negative). The primary outcome was early PPH which was further stratified by clinical severity according to the decrement in hemoglobin concentration after delivery. Categorical variables were compared using the χ2 test while continuous variables were compared using the student's t-test. All data were further analyzed using a stepwise logistic regression model.

**Results:**

1,594 (35.3%) of 4,516 women included in this analysis had O blood type. Early PPH occurred in 44 women (2.7%) with O blood type and 65 women (2.22%) with other blood types. O blood type was not associated with an increased risk for early PPH (OR 1.24, 95% CI 0.84–1.82, P = 0.275). This lack of association remained unchanged after stratification by PPH severity. There was also no significant association between Rh blood type and the risk for early PPH (OR 0.97, 95% CI 0.44–1.4, P = 0.422).

**Conclusions:**

In this cohort, O blood type was not associated with an increased risk for early PPH.

## Introduction

Postpartum hemorrhage (PPH) remains the most common cause for maternal death and one of the major complications of the puerperium [1]. It is defined as the occurrence of blood loss of at least 1000mL following birth that continues despite the use of initial measures including first line uterotonic agents and uterine massage [2]. Early PPH is defined as bleeding that occurs within 24 hours from birth, while late PPH occurs between 24 hours and 6 weeks postpartum [3]. Identifying pre-delivery risk factors allows for earlier recognition and prevention of PPH [4]. Coagulation disorders are recognized as underlying causes for PPH, with von Willebrand disease (VWD) being the most common hereditary coagulopathy associated with PPH [5]. The von Willebrand factor (VWF) molecule forms an adhesive bridge between platelets and vascular subendothelial structures as well as between adjacent platelets at sites of endothelial injury. In addition, it functions as a carrier of the coagulation protein factor VIII and protects it from early degradation in the bloodstream. Thus, any quantitative or qualitative disturbance in the VWF will result in increased bleeding risk [6]. Unsurprisingly, the incidence of PPH is increased among women with VWD as compared to healthy women [7– 9]. Less commonly discussed, the ABO blood type may influence hemostasis in general and PPH in particular [4, 10, 11]. One of the proposed explanations for this possible association is the interaction between ABO blood type and VWF [12–14]. VWF molecules contain side chain oligosaccharides that also belong to the A and B blood group antigens. The presence of these antigens reduces the clearance of VWF molecules from the body. As a result, individuals with A, B, and AB blood types have VWF levels approximately 25–30% higher than those with the O blood type [12–14].

The current data regarding the association between blood type and PPH is conflicting [4, 10, 11]. One prospective study including 304 obstetric patients found no increased risk for PPH in O blood type carriers after operative and non-operative deliveries (OR 0.81, 95% CI 0.6–1.08) [10]. Another retrospective study including 125,768 parturients found that women with O blood type had an increased risk for PPH, as well as for a postpartum decrement in hemoglobin concentration of 2 to 4 gr/dL but not of 5–7 gr/dL [4]. A recent study including 1,487 women reported on larger postpartum blood loss among women with O blood type as compared to women with other blood types [11]. In view of these conflicting data, the aim of the current study was to further investigate the association between O blood type and the risk for early PPH in a cohort of Israeli women who gave birth at a university-affiliated tertiary medical center.

## Methods

After obtaining the approval of the Institutional Review Board (IRB) Committee for Human Subjects (Approval No. 0094-16-CMC), de-identified data was obtained from the Chameleon system database for women who gave birth at Carmel Lady Davis Medical Center in Haifa, Israel between December 1, 2014 and March 31, 2016. Due to the retrospective nature of this study, the IRB committee waived the requirement for patients’ informed consent. Carmel Medical Center is a university-affiliated tertiary hospital with a large obstetric unit serving both Jewish and Arab populations from the city of Haifa and vicinity. Women aged 20–45 years, giving birth between 34 to 42 weeks of gestation and whose ABO blood type was known and registered in the computerized database were included in the study. Data collected from each woman included age, height, weight, major (A, B, AB, O) and minor (Rh) blood types, hemoglobin concentration at admission and postpartum, pregnancy number, birth number, pregnancy week, number of fetuses, birth type (spontaneous, instrumental, or cesarean), uterine revision (manual removal of retained placental tissue from the uterus following delivery), administration of oxytocin for induction or augmentation of labor, diagnosis of PPH and postpartum or intrapartum blood products transfusion. Women were categorized into O and non-O (A, B, or AB) blood types. The primary outcome was early PPH. In our institution PPH is defined as having one or more of the following criteria: (1) increased postpartum bleeding leading to hypovolemic symptoms such as hypotension, tachycardia, or oliguria; (2) estimated blood loss over 500 mL with vaginal birth or over 1,000 mL with cesarean delivery; (3) more than 10% decrement in hematocrit after delivery; and (4) the need for blood transfusion [15]. A diagnostic code for PPH is routinely entered into the medical chart of every woman who fulfills at least one of these criteria. Women with known coagulation disorders including VWD, those who took anticoagulation medications, those with an unknown blood type, intrauterine fetal death, late PPH, uterine rupture, placenta accreta and fibroid uterus were excluded from the study. Women with PPH were further stratified according to hemoglobin decrement postpartum. This decrement was calculated by comparing the lowest hemoglobin measured postpartum to that measured upon admission to the hospital. Sample size estimation was based on a presumed prevalence of the O blood type of 35%. Based on previously reported data, the prevalence of PPH was assumed to be 3.5% and 2% among O and non-O blood type carriers, respectively [16– 19]. To achieve a power of 80% with a level of significance of 0.05 and a sampling error of 2%—a sample of 3,700 women was required for this study. Data analysis was performed using SPSS software version 22. Comparisons of categorical variables were carried out using the χ2 or Fischer exact tests, while those of continuous variables were done using the student's t-test. Relevant data were further processed using a stepwise logistic regression model to estimate the net effect of each variable on the final statistical outcome. P < 0.05 was considered as statistically significant for all comparisons.

## Results

**Fig 1:**
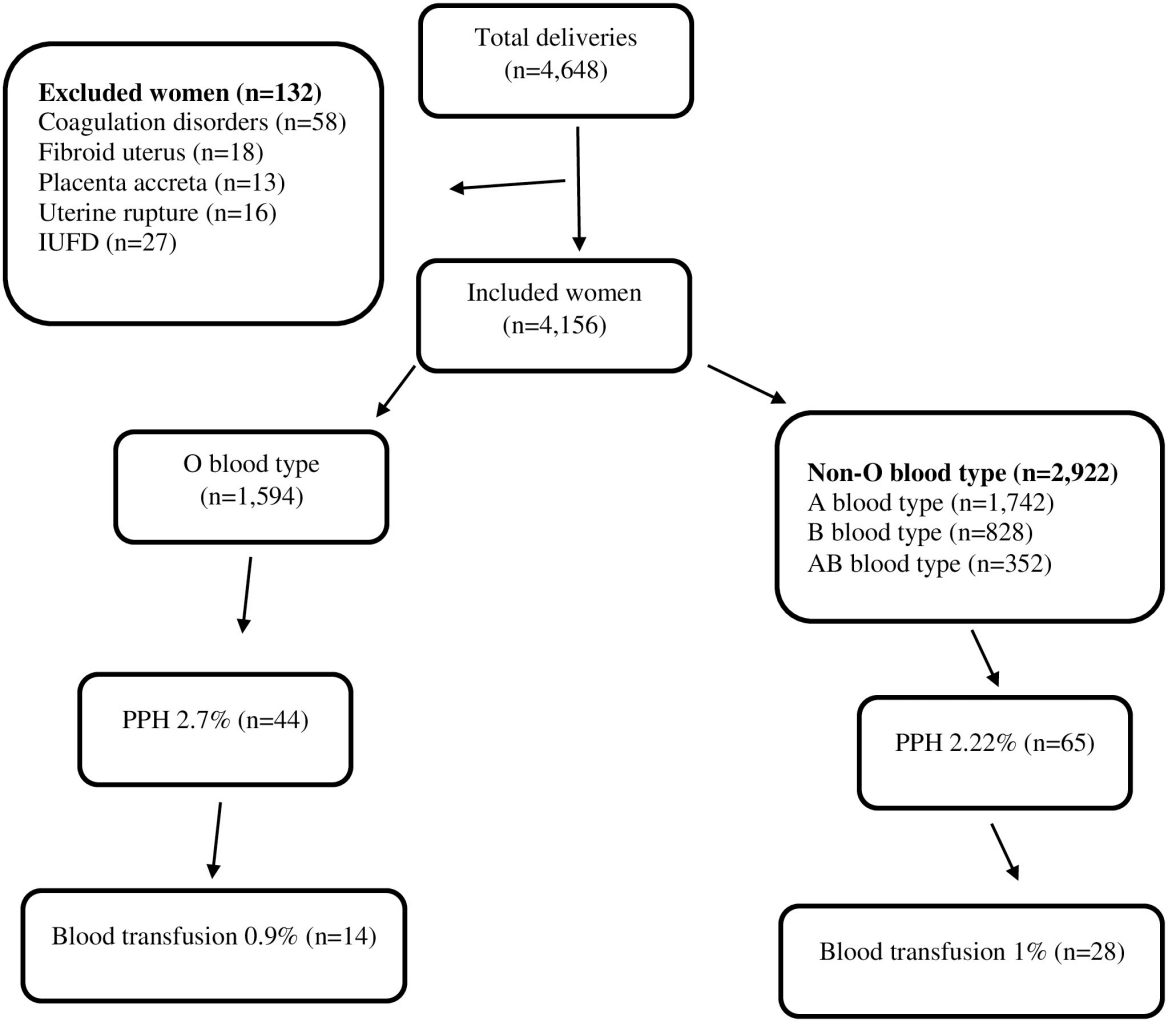
Patients' recruitment flow chart.

Four thousand six hundred and forty eight women delivered during the study period. Fifty eight women were excluded from the study for having coagulation disorders or for taking anticoagulation medications (Fig 1). Eighteen women were excluded for having a fibroid uterus, 13 for having placenta accreta, 16 for uterine rupture and 27 for intrauterine fetal demise. The final cohort included 4,516 women who met the inclusion criteria. One thousand five hundred and ninety four women (35.3%) had O blood type, 1,742 (38.6%) had A blood type, 828 (18.3%) had B blood type and 352 (7.8%) had AB blood type. Mean age of the study population was 32.0 ± 4.8 years. Mean height was 163 ± 6 cm, and mean weight was 74.3 ± 13.3 kg. The median number of previous births was 2, and the mean gestational age at birth was 39 ± 1.9 weeks. One hundred and twenty one (2.6%) women had twin pregnancies, 1,144 (25.3%) underwent cesarean deliveries, 250 (5.5%) underwent vacuum extraction and 668 (14.8%) had a history of a previous cesarean delivery. Nine hundred and thirteen women (20.2%) received oxytocin for induction or augmentation of labor. Perineal tears grades I, II, III, and IV were diagnosed in 13.4%, 17.01%, 0.64% and 0.02% of all deliveries, respectively. The average hemoglobin level at admission was 12.2 g/dL.

Compared to women with other blood types, those with O blood type were slightly younger (Table 1). The two groups were otherwise comparable for all demographic and clinical characteristics. Early PPH was found in a total of 109 women (2.4%) of whom 42 (39%) received blood transfusion during labor or during hospitalization. According to a univariate analysis, there was no statistically significant difference in the prevalence of PPH, or in the need for blood transfusion between women with O and non-O blood types (OR 1.24, 95% CI 0.84–1.82, P = 0.275 and OR 0.91, 95% CI 0.4–1.7, P = 0.789, respectively) (Table 1). Stratification by postpartum hemoglobin decrement did not change this observation (Table 2). The Rh sub-type (positive vs. negative) did not have an effect on these outcomes (OR 0.97, 95% CI 0.44–1.4, P = 0.422). Factors associated with increased risk for early PPH included nulliparity (OR 1.35, 95% CI 1.10–1.66, P = 0.004), vacuum extraction (OR 3.0, 95% CI 1.7–5.3, P < 0.0001), and oxytocin administration for induction or augmentation of labor (OR 2.25, 95% CI 1.5–3.3, P < 0.0001) (Table 3). Using a stepwise multiple logistic regression model, intrapartum oxytocin administration and vacuum extraction remained the most significant variables affecting the risk for early PPH (OR 2.04, 95% CI 1.35–3.08, p = 0.001 and OR 2.49, 95% CI 1.37–4.5, p = 0.003, respectively) S1 Table.

**Table 1:** Comparison of demographic and clinical characteristics of women by ABO blood type.

	Blood type		
	O (N = 1,594)	A,B,AB (N = 2,922)	P value *
Age (years)	31.8 ± 4.9	32.1 ± 4.8	0.039
Height (cm)	163 ± 0.06	163 ± 6	0.393
Weight (kg)	74.2 ± 13.4	74.4 ± 13.2	0.565
Pregnancy number	2 (1–3)	2 (1–3)	0.215
Birth number	2 (1–3)	2 (1–3)	0.208
Preterm birth (< 37 weeks)	108 (6.7%)	207 (7%)	0.697
Birth type			0.279
Cesarean	383 (24%)	761 (26%)	
Vacuum	94 (5.9%)	156 (5.3%)	
Vaginal	1,117 (70.1%)	2,005 (68.6%)	
Twin pregnancy	44 (2.8%)	77 (2.6%)	0.803
Previous cesarean section	226 (14.2%)	442 (15.1%)	0.400
Oxytocin administration	335 (21%)	578 (19.8%)	0.323
Baseline hemoglobin level (gr/dL)	12.28 ± 0.89	12.17 ± 0.92	0.9
Perineal tears (degree)			
0	1,075 (67.4%)	2,035 (69.6%)	
1–2	512 (32.1%)	863 (29.5%)	
3–4	7 (0.4%)	24 (0.8%)	
Total	519 (32.6%)	887 (30.4%)	0.126
Uterine revision	22 (1.4%)	36 (1.2%)	0.673
Placental abruption	29 (1.8%)	54 (1.8%)	0.945
Preeclampsia	39 (2.4%)	86 (2.9%)	0.311
Early PPH	44 (2.7%)	65 (2.22%)	0.275
Early PPH with blood transfusion	14 (0.9%)	28 (1%)	0.789

Data are presented as number (percent), mean ± SD, or median (range).

* Calculated using the χ2 test for categorical variables and student's t-test for continuous variables.

**Table 2:** Postpartum hemoglobin decrement among women with PPH according to ABO blood type.

	Blood Type		
Hemoglobin Decrement (g/dL)	O (N = 1,594)^#^	A,B,AB (N = 2,922) ^†^	P value *
0–1	3 (0.18)	3 (0.10)	0.43
1.1–2	4 (0.25)	4 (0.13)	0.46
2.1–3	7 (0.43)	14 (0.47)	1
3.1–4	6 (0.37)	12 (0.41)	1
4.1–5	13 (0.81)	13 (0.44)	0.148
5.1–6	8 (0.50)	9 (0.30)	0.31
≥ 6.1	2 (0.12)	7 (0.23)	0.5

Data are presented as number (percent).

* Calculated using χ2 test.

^#^ Data unavailable for one patient

^†^ Data unavailable for 4 patients.

**Table 3:** Factors associated with early postpartum hemorrhage.

	Univariate analysis				Multivariate analysis	
	Early PPH (N = 109)	No early PPH (N = 4,407)	OR (95% CI)	P value*	OR (95% CI)	P value^#^
Age (years)	32.2 ± 4.9	31.98 ± 4.9	1.00 (0.97–1.05)	0.641		
Height (cm)	163 ± 6	163 ± 6	0.14 (0.007–2.8)	0.199		
Weight (kg)	73.4 ± 14.2	74.3 ± 13.3	0.99 (0.98–1.01)	0.565		
Pregnancy number (Gravida)	2 (1–3)	2 (1–3)	0.89 (0.78–1.03)	0.120		
Birth number (Parity)	1 (1–2)	2 (1–3)	1.35 (1.10–1.66)	**0.004**	1.23 (1.00–1.51)	0.052
Birth type						
Cesarean delivery	29 (26.2)	1,115 (25.3)	1.22 (0.79–1.9)	0.373		
Vacuum delivery	15 (13.7)	235 (5.3)	3.0 (1.7–5.3)	**<0.0001**	2.49 (1.37–4.51)	**P = 0.003**
Twin pregnancy	3 (2.7)	118 (2.6)	1.03 (0.32–3.3)	0.962		
Previous cesarean delivery	16 (14.6)	652 (14.8)	0.99 (0.58–1.69)	0.972		
Intrapartum oxytocin administration^†^	39 (35.7)	874 (19.8)	2.25 (1.5–3.3)	**<0.0001**	2.047 (1.35–3.08)	**P = 0.001**
Baseline hemoglobin level (gr/dL)	11.89 ± 5.7	12.2 ± 5.6	0.97 (0.94–1.00)	0.050		
Perineal tears						
Grade 1–2	30 (27.5)	1,345 (30.5)	0.88 (0.57–1.3)	0.552		
Grade 3–4	2 (1.8)	29 (0.65)	2.7 (0.64–11.6)	0.177		
Placental abruption	2 (1.8)	81 (1.8)	1.00 (0.24–4.1)	0.998		

Data are presented as number (percent), mean ± SD, or median (range).

* Calculated using the χ2 test for categorical variables and student's t-test for continuous variables.

^#^ Performed using a multiple logistic regression model adjusting for the following variables: Blood type, birth number, birth type and oxytocin administration.

^†^ For induction or augmentation of labor.

## Discussion

In this cohort of healthy women with no known coagulopathies, delivering at 34–42 weeks of gestation at the Carmel Medical Center in Haifa, there was no significant association between blood type and the risk for early PPH. The incidence of PPH found in our study is consistent with previous data [4, 16, 17]. Blood type distribution in the study population corresponds to that previously reported in the Israeli population with 35% of women carrying the O blood type [20].

Additional findings were that administration of oxytocin for induction or augmentation of labor and vacuum extraction were the most significant risk factors for early PPH. These findings are consistent with previously reported data showing higher morbidity (including PPH) with oxytocin administration, especially following prolonged exposure [21– 24]. This may be attributed to a higher risk for postpartum uterine atony and labor dystocia related to this mode of treatment [21]. Prolonged and high-dose oxytocin exposure can cause desensitisation of the oxytocin receptors in the uterine smooth muscle, thereby limiting further oxytocin-mediated contraction responses which may result in postpartum uterine atony [24]. The association between vacuum extraction and early PPH has also been reported previously, even when compared to cesarean delivery [25]. This may be attributed to a higher risk for vulvar, perineal, vaginal and cervical lacerations as well as for uterine atony and even rupture [26, 27].

To date, only three studies have evaluated the association between O blood type and the risk for PPH [4, 10, 11]. A prospective study by Clark et al. enrolled 4,157 unselected pregnant women in Glasgow, Scotland between 1997 and 2000. Factor V Leiden (FVL) analysis was uniformly performed between 7 and 16 weeks of gestation, and all women were followed throughout labor and delivery and up to 6 weeks postpartum. Peripartum hemorrhage was defined as any bleeding episode visually assessed as 500 mL or more irrespective of the need for clinical intervention. One hundred forty two women (3.61%) were found to be FVL carriers, 52.4% had O blood type and 47.6% had non-O blood types. One hundred ninety six women developed peripartum hemorrhage following non-operative and 105 following operative delivery. The O blood type was not associated with an increased risk for intrapartum hemorrhage as compared to non-O blood types (OR 1.00, 95% CI 0.82–1.2 for non-operative deliveries and OR 0.82, 95% CI 0.59–1.15 for operative deliveries) [10]. Another large retrospective cohort study conducted by Drukker et al. obtained data from 125,768 women delivering beyond 24 weeks of gestation in a single institution in Jerusalem, Israel between 2005 and 2014, including both vaginal and cesarean deliveries. Exclusion criteria for this study were any missing blood type or hemoglobin data as well as intra-uterine fetal demise. PPH was defined as an estimated blood loss of 500 mL or more after vaginal delivery or 1000 mL or more following cesarean delivery. Women with O blood type were found to have a mildly increased risk for PPH (2.3% vs. 2%, p = 0.001), as well as for a postpartum decrement in hemoglobin concentration of 2–4 gr/dL (P < 0.001) but not of 5–7 gr/dL as compared to women with non-O blood types. These findings remained valid after a multivariate analysis [4]. A recent prospective cohort study conducted by Kahr et al. enrolled 1,487 parturients undergoing both vaginal and cesarean deliveries. Blood loss was estimated by visual assessment and by comparing pre- and postpartum hemoglobin concentrations. The O blood type was found to be associated with a minor increase in postpartum blood loss (529.2 mL ± 380.4 mL vs. 490.5 mL ± 276.4 mL, P = 0.024). This association remained statistically significant after a multivariate analysis, incorporating relevant variables such as uterine atony, placenta previa and adherent placenta [11]. In our study, no association was found between major or minor blood types and the risk for early PPH or the need for blood transfusion. These results agree with Clark's but contrast with Drukker's and Kahr's results. Stratifying PPH severity by postpartum hemoglobin decrement, similar to Drukker’s approach, did not change our findings. The discrepancies between our study results and those of Drukker and Kahr may be explained by the different populations recruited and by the different definitions for PPH applied in each study. In our study, unlike Drukker's and Kahr's, women with known coagulopathies and those taking anticoagulant therapy were excluded. Furthermore, Kahr's study did not assess PPH prevalence as a primary outcome but rather evaluated total peripartum blood loss. Moreover, the minor difference of 39 mL between the O and non-O blood types found in his study may not necessarily be of clinical significance. This is supported by the lack of association between the O blood type and postpartum hemoglobin decrement reported in this study. Similarly, Drukker's study demonstrated only a minor increase in the prevalence of PPH among O blood type carriers (2.3% vs. 2.0%), the clinical significance of which warrants further investigation.

Several studies have reported that the ABO blood type system influences hemostasis, including in the context of thromboembolic events during pregnancy and postpartum. A case-control study conducted by Jick el al. in 1969, reported a relative risk of 2.1 for venous thromboembolism (VTE) (95% CI 1.5–3.1) among pregnant women with non-O blood types as compared to women with the O blood type [28]. Another study reported by Talbot et al. in 1970 in a cohort of pregnant British women found a relative risk of 1.7 (95% CI 1.1–2.6) for VTE among non-O blood type carriers [29]. A case-control study published by Larsen et al. in 2005 including 71,729 women found an increased risk for VTE in AB (OR 2.2 and 2.7 during pregnancy and puerperium, respectively), and A (OR 3.9 and 2.4, respectively) but not B (OR 1.5 and 1.0, respectively) blood type carriers, which remained statistically significant after adjustment for potential confounding factors such as age, smoking, parity, history of diabetes mellitus and body mass index [30].

In the general population, a meta-analysis by Dentali *et al*. including 22 studies conducted between 1946 and 2012, the O blood type was found to be a significant risk factor for bleeding [18]. In contrast, other studies found no association between the O blood type and bleeding tendency with regards to intracranial hemorrhage in neonates and adults or in the setting of abdominal and pelvic surgeries [31– 35].

### Strengths and limitations

Our study's main strengths include a large sample size of over 4,000 deliveries with robust demographic, medical and obstetric data which were entered electronically in real-time and can therefore be assumed to be correctly documented. Its limitations include a retrospective design as well as the inherent difficulty to diagnose PPH, as this diagnosis relies on subjective estimation of blood loss, rather than on quantitative measurements, although recent protocols include quantitative blood loss measurements (QBL), this was not available in our database. Data regarding specific indication for oxytocin administration (i.e. induction or augmentation of labor), its dosage, timing and duration of administration were not available in our study, which is unfortunate since oxytocin use was found to be associated with our primary outcome of PPH, and we cannot differentiate between induction of labor with oxytocin or labor augmentation, nor can we determine whether prolonged use is what resulted in PPH. Furthermore, we did not measure VWF levels, and these increase during the third trimester of pregnancy and continue to do so after delivery and under stressful conditions [36], and cannot describe the possible interaction between VWF levels and blood type (specifically blood type O) in our cohort. These physiological changes might have obscured the association between blood type and bleeding tendency since minor differences in VWF levels between different blood types might have been eliminated.

In view of the paucity and inconsistency of the existing data, we suggest that the role of blood type in the pathophysiology of obstetric and gynecologic hemorrhagic disorders should be further investigated in larger cohorts of patients. Plasma VWF levels may add valuable information and should probably be assessed concomitantly in all patients. If such a role indeed exists, strategies should be developed to reduce the incidence and improve the therapeutic options for these disorders in high risk populations.

## Supporting information

S1 Table Dataset of subjects. (XLSX)
